# Assessing the knowledge, attitudes and practice of type 2 diabetes among patients of Saurashtra region, Gujarat

**DOI:** 10.4103/0973-3930.54288

**Published:** 2009

**Authors:** Viral N. Shah, P. K. Kamdar, Nishit Shah

**Affiliations:** Department of Medicine, Govt. Medical College, Bhavnagar, Gujarat, India; 1 Department of Medicine, M. P. Shah Medical College, Jamnagar, India

**Keywords:** Knowledge, attitude and practice study, type 2 diabetes

## Abstract

As India will ranks first in diabetes now and will continue to do so in 2025, we must prevent the disease by various measures. Before setting the programmes, we should have ample data on the population's knowledge, attitude and practice (KAP) of diabetes. There are some epidemiological studies from southern India, Mumbai and north India, but there is no KAP study from Gujarat. The study was conducted between the period of June 2007 to November 2007 at three centers of Saurashtra region. Out of 300 patients who were given questionnaire, 238 patients were included for the analysis, rest were excluded due to various reasons. 52% were male. Mean age of patients was 55.82±10.2 years (95% CI 54.5-57.1) with mean weight of 64.52±10.96 Kg (95% CI 62.8-66.2). Mean duration of diabetes was 8.2±6.8 years (95% CI 7.2-9.1). 46% of patients knew the pathophysiology of diabetes. Nearly 50% knew the complications of diabetes. Dietary modifications were relied more than exercises among the interviewed subjects. Most of the lacunae in knowledge prevailed in drug therapy of diabetes. Insulin was not favored by most of patients. An encouraging finding in our study was that most believed in self-care and ready to change. Consultation time given by their treating doctors was less than 5 minutes in nearly 50%. Foot care and education to prevent complications were least suggested by doctors.

## Introduction

Prevalence of type 2 diabetes is increasing globally,[1] more so in developing countries like India due to rapid urbanization.[2,4] It is estimated that prevalence of diabetes will rise to 5.5% in 2025 as compared to 4% in year 1995.[3] The total direct cost for diabetes management has doubled from 1998 to 2005.[5] Therefore, prevention is important both on monetary and human matters. There is an increasing amount of evidence that the patient education is the most effective way to lessen the complications of diabetes and its management.[6] Education is likely to be effective if we know the characteristic of the patients in terms of knowledge, their attitude and practices about diabetes. There are numerous studies with special emphasis on epidemiology mainly from South India, Delhi and Mumbai.[7,8] However, despite our extensive literature search we could not find any KAP study of diabetes mellitus especially from the Saurashtra region.

## Aims

The aim of the study was to assess the knowledge, attitude and practice of patients with type 2 DM in Saurashtra region.

## Materials and Methods

The study was conducted between the period of June 2007 to November 2007 at three centers 1) G.G.Hospital, Jamnagar 2) Sir Takhatsinhji Hospital, Bhavnagar 3) S. J. Eye Hospital, Gondal, Rajkot. All these places belong to Saurashtra region, Gujarat. First two are tertiary care hospitals with teaching facilities and third is private sector in rural area run by a Non Government Organization (NGO). The patients attending out-door facility of these hospitals were included in the study and given predesigned proforma to fill the questionnaire. To avoid bias, the patients who were not being treated by authors at the time of this study were only included. The questionnaire did not contain any questions which can reveal the identity of patients or their treating doctors. Out of 300 patients who were given questionnaire, only 238 patients were included for the final analysis. Rest were excluded due to incomplete or irrelevant information and poor handwriting. Data were analyzed using Microsoft Excel 2006.

## Results

### Patients characteristic and demographic profile

A total of 238 (79.33%) patients out of 300 were included for final analysis. Out of these 238, 120 (50.42%) were males. 178 (74.78%) were Hindu by religion, 53 (22.26%) were Muslim. 192 (80.27%) cases were from urban area while 46 (19.32%) were from rural area. Age wise distributions of all patients were shown in Table 1. Mean age of patients was 55.82±10.2 years (95% CI 54.5-57.1) with mean weight of 64.52±10.96 Kg (95% CI 62.8-66.2). Mean duration of diabetes was 8.2±6.8 years (95% CI 7.2-9.1). Detail characteristics like occupation, family income and education, and their treating doctor are shown in Table 2.

**Table 1:** Age wise distribution of patients (n=238)

Age (years)	No of patients (%)
30-39	9 (3.78)
40-49	50 (21.08)
50-59	96 (40.33)
60-69	59 (24.78)
70-79	20 (8.40)
> 80	4 (1.68)

**Table 2:** Characteristics of patients with type 2 diabetes (n=238)

Patients characteristics	Percentage
Occupation	
Students	3.8
Housewife	43.90
Service	17.46
Laborer	35.77
Annual income	
< 20,000	39.47
Up to 50,000	27.19
>50,000	33.33
Treating doctor	
General practitioner	17.69
Physicians	79.20
Endocrinologist	3.07
Source of information	
Doctor	76.44
Media	4.44
Another persons	19.11
Internet	
Educations	
Illiterate	36.64
Up to school	52.35
Graduation	10.99

### Knowledge regarding diabetes

**Figure 1:**
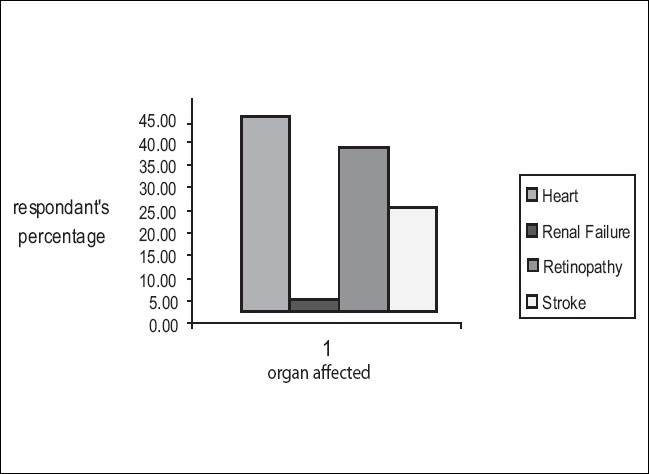
Frequency distribution of knowledge regarding complications of diabetes

Despite 8 years of average duration of diabetes, about 46% of patients knew the pathophysiology of diabetes. Fewer (38.23%) still believed that diabetes can be cured. Many other aspects are covered in Table 3. Among the knowledge of complications of diabetes, renal complication was least known to patients. Most were afraid of heart related complications. Details of knowledge of complications depicted in Figure 1. Table 4 shows the responder knowledge regarding diet and exercises. Dietary modifications were relied more than exercises among the evaluated patients. Most of the lacunae in knowledge prevailed in drug therapy of diabetes. Insulin was disfavored by most of patients. Details of knowledge of drug therapy in diabetes are shown in Table 5.

**Table 3:** Frequency distribution of respondent's knowledge regarding etiology and features diabetes

Questions to assess knowledge	Correct answers (%)
What is diabetes?	46.63
What causes diabetes?	17.64
Can diabetes be cured?	38.23
How can diabetes be detected?	82.77
Features of diabetes:	
Polyuria	58.82
Can be Asymptomatic	5.42
Weight loss	2.52
Increase hunger	5.88
Recurrent infection	43.28
Is diabetes hereditary?	57.98
Is diabetes infectious?	5.042

**Table 4:** Percentage of respondent's knowledge regarding exercise and diet in diabetes

Knowledge of exercises and diet in diabetes	Percentage
What should be done to control DM?	
Exercises	51.23
Dietary modifications	74.78
Stop smoking/alcohol	7.14
Is exercise beneficial?	83.16
Exercises should be done by only obese person?	84.05
Bitter substances can cure DM	53.37

**Table 5:** Frequency distribution of respondent's knowledge of drug therapy in diabetes

Once DM is controlled drugs should be stopped	22.26%
Drug is more important than diet control	51.26%
Insulin is to be avoided as far as possible	48.31%
Herbal drugs are better	39.49%
Insulin is habit forming	51.68%

### Attitude and practices in diabetes

An encouraging part in our study was that most believed in self care in diabetes. Majority of the patients could not afford a blood glucose meter and hence, could not check their blood glucose level regularly. Practice of taking herbal drugs prevailed in approximately 40% of patients. Table 6 details the attitude and practices in our patients.

**Table 6:** Frequency distribution of respondent's attitude and practice toward diabetes

Respondent's attitude and practice	Percentage
Who is responsible for DM care?	
Your self	65.12
Doctor	39.07
Family	34.03
Do you include fruits in your diet regularly?	54.21
Do you take green leafy vegetables in diet?	31.93
Do you have glucometer?	10.08
Do you check your sugar regularly?	70.16 (monthly)
Do you check your foot regularly?	56
Do you take herbal drugs?	38.65

### Appraisal of doctor by patients

Most patients were dissatisfied with the consultation time given by their treating doctors. The time given was less than 5 mins in nearly 50%. Foot care and watch for complications were least suggested by doctors [Table 7].

**Table 7:** Frequency distribution of respondent's appraisal of their treating doctors

How much time your doctor devotes to you?	
Five minutes	43.27
Ten minutes	26.89
More than 10 minutes.	10.8
Your doctor explains you diabetes thoroughly?	85.29
Does you doctor explain diet?	85.29
Does your doctor explain exercises?	64.28
Does your doctor check feet regularly?	34.43
Does your doctor motivate you for self care in diabetes?	33.61

## Discussion

Most studies regarding epidemiology and prevalence of diabetes were conducted from south India[9–12] and very few studies from north India.[13] There is no study regarding KAP is available for Saurastra region and hence, this study is in attempt to gather the data regarding the same.

Since the study conducted among patients with type 2, average age was between 50-59 years (40.33%) and average duration of diabetes among patients was 8.2years. nearly 60% of patients were from low socio-economical status and 36% were illiterate. Since the majority of patients were recruited from government-run hospitals, there may be bias regarding these two factors. Both affordability and literacy may be the problem in diabetes education and management. Education of vulnerable communities can become a cost-effective public health strategy. It has been shown that self-care among individuals with type 2 diabetes improved glycemic control[14] and reduced complications.[15]

American Diabetic Association has defined self management education as the process of providing the person with diabetes the knowledge and skill that is needed to perform self care, manage crises and make life style changes. National standard for self care management in diabetes has been set by Mensing[16]*et al*. To achieve such stanted self care patients and doctor should work together. There is emphasis on teaching pathophysiology and its relation with treatment, nutritional aspects, medications, complications, goal setting and psychosocial adjustments. Considering these standards, we formulated our questionnaire.

During evaluation of knowledge part, we found that most patients didn't know what diabetes is (63%) and what the consequences of diabetes are in the long run (nearly 60%). Our respondents only few knew that a common complications was heart attack. Another crucial finding of our study was limited knowledge of complications and importance of life style modification.

Three main findings of our study which may be responsible for low diabetes education among the patients were: 1) Nearly 40% of patients were below poverty line and hence could not afford even minimum standard care and therapy. 2) Only 3% of patients were treated by endocrinologists. This is because very few endocrinologists are available in Gujarat. None of them are available in government run hospital and, patients can not afford the private care. No single institute in Gujarat had superspeciality course on diabetology or endocrinology. There are a number of studies which showed that treatment by a diabetes specialist improves treatment outcomes compared to treatment by a generalist.[17–22] 3) Most important factor is low level of education, only 10% were graduate and nearly 37% were completely illiterate. Therefore, illiteracy may be the most important obstacle in diabetes management of such patients. It is rightly said that education of vulnerable communities can become a cost-effective public health strategy.[23]

Our study showed that people in Saurashtra region wrongly believe that diabetes can be cured with bitter substances and allopathic drugs are harmful to the body. They also have many misconceptions about insulin.

An encouraging part of the study is that although there was, quite low diabetes self education among patients, most of patients believed that they are responsible for their care and this implies that they were ready to change if motivated or educated properly.

Though it was not the aim of study, we studied the appraisal of doctors by their patients. The shocking fact was physicians could spare very limtied time for their patients and a search for complications was ignored by most. Foot care checking and self care motivation, the two main aspects of diabetes care were ignored by most of the treating practitioners. Though there are few studies regarding the cost analysis of diabetes in India, it is a proven fact that ignoring such vital aspects may increase cost steeply.[24] There are Indian standards set by Agrawal[25] and others on how to set up a diabetes clinic. There should be emphasized to strengthen the diabetic care.

### Recommendations

- Emphasis on improving the literacy rate of the population.
- To increase the availability of endocrinologists.
- Diabetes education must be imparted by every clinician as per standard norms.
- Generalist or primary care physician should be enriched with more knowledge by CME and other programmes.
- Media and Non Government Organisation should be involved in the daunting task of removing misbelieves, ignorance and instituting diabetes preventive measures in the community.
